# Comparison of the effects of remimazolam and dexmedetomidine on early postoperative cognitive function in elderly patients with gastric cancer

**DOI:** 10.3389/fnagi.2023.1123089

**Published:** 2023-06-05

**Authors:** Yong Qing Liao, Jia Min, Zheng Xia Wu, Zhi Hu

**Affiliations:** Department of Anesthesiology, Medical Center of Anesthesiology and Pain, The First Affiliated Hospital of Nanchang University, Nanchang, Jiangxi, China

**Keywords:** cognitive function, remimazolam, dexmedetomidine, elderly, gastrectomy for gastrointestinal cancer

## Abstract

**Purpose:**

To compare the effects of remimazolam and dexmedetomidine on early postoperative cognitive dysfunction (POCD) in aged gastric cancer patients.

**Methods:**

From June to December 2022, 104 elderly patients (aged 65–80 years) received laparoscopic radical resection of gastric cancer at the First Affiliated Hospital of Nanchang University. Using the random number table approach, the patients were separated into three groups: remimazolam (Group R), dexmedetomidine (Group D), and saline (Group C). The primary outcome was the incidence of POCD, and secondary outcomes included TNF-α and S-100β protein concentrations, hemodynamics, VAS scores, anesthesia recovery indicators, and the occurrence of adverse events within 48 h postoperatively.

**Results:**

At 3 and 7 days after surgery, there were no statistically significant differences in the incidence of POCD, the MMSE and MoCA scores between groups R and D (*p* > 0.05). However, compared to the saline group, both groups had higher MMSE and MoCA scores and decreased incidences of POCD. These differences were statistically significant (*p* < 0.05). Between group R and group D, there were no statistically significant changes (*p* > 0.05) in the levels of TNF-α and S-100β protein at the three time points (at the end of the surgery, 1 day later, and 3 days later). Even though neither group’s concentration of the two factors was as high as that of the saline group, the differences were statistically significant (*p* < 0.05). At all three time points—following induction (T_2_), 30 min into the operation (T_3_), and at the conclusion of the surgery (T_4_)—the heart rate and blood pressure in group R were greater than those in groups D and C. Statistics showed that the differences were significant (*p* < 0.05). The incidence of intraoperative hypotension was highest in group D and lowest in group R (*p* < 0.05). The dose of propofol and remifentanil, group C > group R > group D. Extubation and PACU residence times did not differ statistically significantly (*p* > 0.05) between the three groups. There was no significant difference in VAS scores between groups R and D after 24 h postoperatively (*p* > 0.05), although both had lower scores than group C, and the difference was statistically significant (*p* < 0.05). The VAS scores between the three groups at 72 h (T_6_) and 7 days (T_7_) were not statistically significant (*p* > 0.05). Adverse reactions such as respiratory depression, hypotension, bradycardia, agitation, drowsiness, and nausea and vomiting had the lowest incidence in group R and the highest incidence in group C (*p* < 0.05).

**Conclusion:**

Remimazolam is similarly beneficial as dexmedetomidine in lowering the incidence of early POCD in aged patients after radical gastric cancer resection, probably due to reduced inflammatory response.

## 1. Introduction

Gastric cancer is one of the most prevalent malignant tumors of the digestive system in China, with the third highest incidence rate of all malignant tumors (Siegel et al., 2021). According to authoritative epidemiological studies, with the emergence of aging social problems in China, the incidence rate of elderly patients with stomach cancer is rising year by year, with incidence and mortality rates of 11.1/100,000 and 8.2/100,000 respectively (Thrift and El-Serag, 2020). Postoperative cognitive dysfunction (POCD) is one of the common complications in elderly patients, which mainly manifests as one or more cognitive deficits that occur after patients undergo surgery, including verbal memory, comprehension, visual memory, visuospatial abstraction, attention, executive function, etc. (Moller et al., 1998). It is difficult to detect but can linger for months, years, or even permanently (Evered and Silbert, 2018). It can harm the patient’s surgical recovery, lengthen hospitalization, diminish the quality of life, and raise mortality (Borges et al., 2017).

The etiology of POCD is complex and the exact mechanism remains unclear. However, the neuroinflammatory response is considered to be the key factor and initiating link (Skvarc et al., 2018). When surgery and anesthesia are performed, the immune system is activated in a nuclear factor-κB (NF-κB)-dependent manner, which results in the release of various pro-inflammatory mediators, including TNF-α, and ultimately leads to a compromised blood–brain barrier and promotes the migration of macrophages into the brain parenchyma, which causes cognitive dysfunction (Subramaniyan and Terrando, 2019).

It has been shown that POCD and Alzheimer’s disease have very similar pathological changes, the deposition of superphosphate Tau protein and amyloidβ (Aβ) in helical filamentous arrangements; the former can cause neuronal dysfunction, while Aβ can induce neuronal inflammatory responses and has neurotoxic effects. The more Aβ deposited in the brain of elderly patients, the more likely they are to develop POCD following surgery (Wang YW et al., 2022).

Dexmedetomidine is a new generation alpha2-adrenoceptor agonist that is widely utilized in the treatment of sedation, analgesia, and anxiety. Dexmedetomidine has been demonstrated to minimize stress response, inflammatory factor production and release (Wang and Wang, 2021), and considerably reduce the incidence of POCD (Li et al., 2021; Wang et al., 2021).

Remimazolam is a novel ultrashort-acting benzodiazepine agonist that works on γ-aminobutyric acid type A (GABA A) receptors to reduce neuronal excitation and thereby achieve hypoactivity and sedation in the body (Kilpatrick, 2021). Remimazolam is metabolized by a nonspecific esterase, and the metabolite has no pharmacological effect, which allows prolonged infusion without accumulation (Zhou et al., 2020). It provides rapid anesthesia and arousal while stabilizing hemodynamics and produces less depression of respiration, making it more suitable for use in elderly and hemodynamically unstable patients (Chen et al., 2021). Remimazolam has been demonstrated to alleviate neuropathic pain, restrict pro-inflammatory factor production (Xie et al., 2021) and decrease cerebral ischemia/reperfusion (I/R) injury (Shi et al., 2022). Furthermore, it reduces oxidative stress and inflammation, to some extent, offers protective advantages for the liver, lungs and brain, supporting the maintenance of cognitive function in the brain (Liu et al., 2021). In addition, patients were sedated with remimazolam during colonoscopy and bronchoscopy, and cognitive function scores showed better neuropsychiatric recovery with remimazolam (Rex et al., 2018; Pastis et al., 2019; Tan et al., 2022).

In summary, we hypothesized that remimazolam might reduce the incidence of POCD in older patients by decreasing intraoperative neuroinflammatory reactions, improving hemodynamic stability, and lowering perioperative opioid usage. The current investigation will contrast the effects of remimazolam and dexmedetomidine on elderly patients undergoing surgery for stomach cancer who experience early postoperative cognitive impairment.

## 2. Materials and methods

### 2.1. Experimental methods

104 elderly patients were enrolled in the First Affiliated Hospital of Nanchang University for laparoscopic radical resection of gastric cancer from June 2022 to December 2022. Non-participants in the study used a computer to generate random numbers, randomly divided into three groups in a 1:1:1 ratio. The generated random sequence numbers were placed in opaque envelopes that were randomly selected and opened by another anesthesiologist who was not involved in the study to reveal the groupings before the patients were admitted to the operating room. To exclude bias due to different surgical practices and habits all patients were from the same group of surgeons. With 34 cases in the remimazolam group (one case developed anaphylaxis intraoperatively and was excluded), 35 cases in the dexmedetomidine group, and 35 cases in the saline group.

Inclusion criteria: (a) aged individuals (65–80 years old) receiving elective laparoscopic radical excision for gastric cancer; (b) American Society of Anesthesiologists (ASA) classification II-III; and (c) BMI 18–24 kg/m^2^.

Exclusion criteria: (a) preoperative Mini-mental State Examination (MMSE) or Montreal Cognitive Assessment (MoCA) cognitive dysfunction (preoperative scores ≤17 for illiterate, ≤20 for primary, ≤22 for secondary, and ≤23 for university); (b) History of cardiac surgery, cerebrovascular accident, and alcoholism; (c) presence of serious organic diseases such as liver and kidney dysfunction; (d) coagulation disorders; (e) severe visual and hearing impairment that prevents communication; and (f) history of psychiatric disorders or long-term sedation or depression medication.

Rejection criteria: (a) extended surgical scope resulting in prolonged surgery time (operative time > 4.5 h); (b) patients who have severe intraoperative bleeding or develop drug allergies; and (c) subjects requesting termination of the test.

The study was authorized by the Ethics Committee of the First Affiliated Hospital of Nanchang University, Ethics Number IIT 2022-089, and patients consented and signed a full informed consent form. The trial was registered at the Chinese Clinical Trials Registry (ChiCTR2300068036). The study protocol followed CONSORT guidelines and was conducted within the relevant guidelines.

A five-lead electrocardiogram (ECG), noninvasive blood pressure (NIBP), heart rate (HR), blood oxygen saturation (SPO2), and depth of anesthesia monitoring (BIS) were all routinely monitored once the patient was brought to the room. Peripheral venous access was opened and radial artery puncture was performed under local anesthesia with 0.8 ~ 1% lidocaine.

Induction of anesthesia: Patients in all three groups were induced with cisatracurium 0.15 mg/kg (Batch Number H20060869, Jiangsu Hengrui Pharmaceutical Co., Ltd.), sufentanil 0.4–0.6 μg/kg (Yichang Renfu Pharmaceutical Co. Ltd., National Drug Standard H20054171) and propofol 1.0–1.5 mg/kg (Xi’an Libang Pharmaceutical Co., Ltd., National Drug Standard H20123318). On this basis, remimazolam mesylate 0.2 mg/kg (Batch Number H20190034, Jiangsu Hengrui Pharmaceutical Co., Ltd.) was added in group R. Patients in group D were given dexmedetomidine 200 μg (Yangzijiang Pharmaceutical Group Co., Ltd., National Drug Standard H20183219) diluted to 50 mL with saline (prepared concentration 4 μg/mL) and pumped 0.5 μg/kg over 10 min before induction of anesthesia. Saline 5 mL was added in group C. After the analgesic, sedative, and muscle relaxant had taken effect, endotracheal intubation was conducted. The PETCO_2_ was maintained at 35–45 mmHg by adjusting the tidal volume and the respiratory rate.

**Figure 1:**
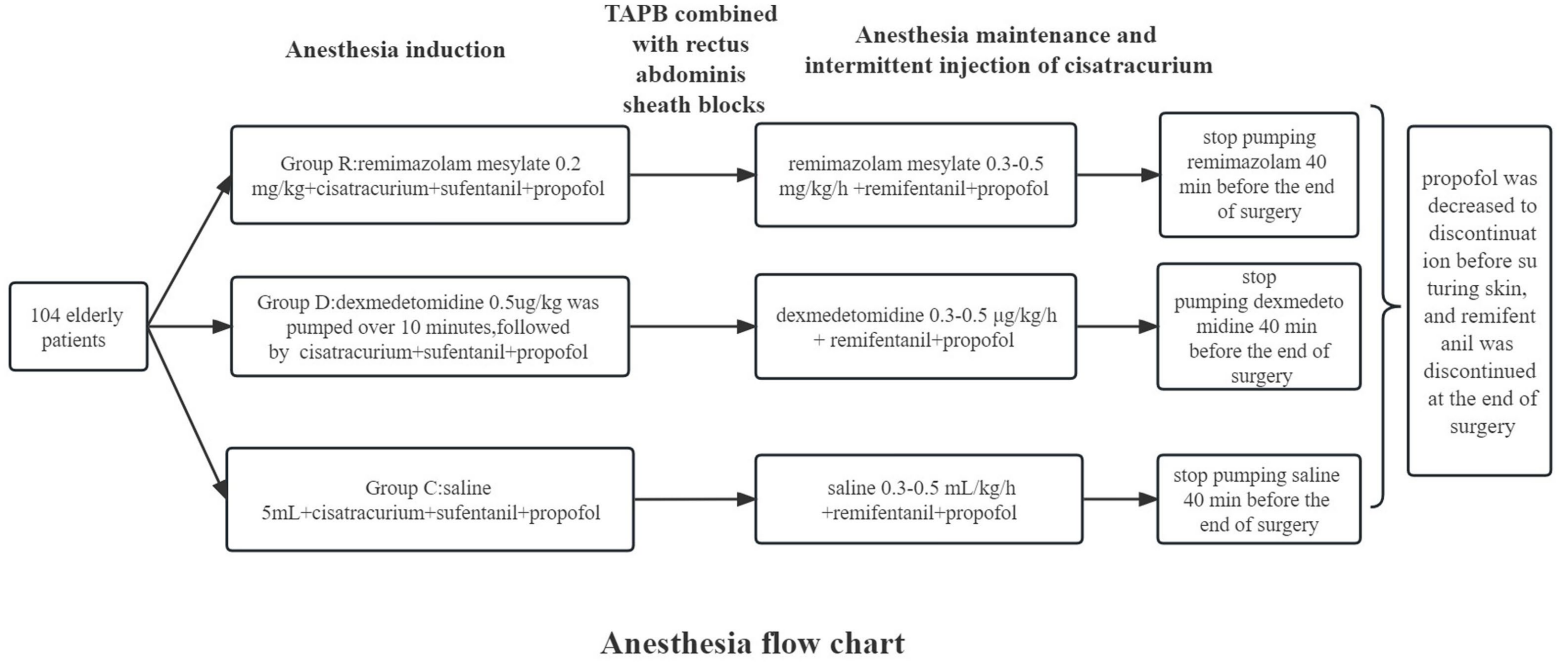
Anesthesia flow chart.

Anesthesia maintenance: In all three groups, propofol 4–8 mg/kg/h + injectable remifentanil hydrochloride 4–8 μg/kg/h (Batch Number H20143315, Jiangsu Enhua Pharmaceutical Co., Ltd.) were pumped intravenously. On this basis, intravenous pumping of remimazolam 0.3–0.5 mg/kg/h was added in group R, stop pumping remimazolam 40 min before the end of surgery; intravenous pumping of dexmedetomidine 0.3–0.5 μg/kg/h was added in group D, and dexmedetomidine was halted 40 min before the conclusion of operation; intravenous pumping of saline 0.3–0.5 mL/kg/h was added in group C, stop pumping saline 40 min before the end of surgery. Cisatracurium 0.1 mg/kg was administered intravenously in all three groups to maintain muscular relaxation. Propofol was decreased to discontinuation before suturing skin, and remifentanil was discontinued at the end of surgery. Intraoperatively, vasoactive drugs were administered according to circulatory fluctuations, BIS values of 40–60 were maintained, and the total amount of propofol and remifentanil was recorded. Following anesthetic induction, all three groups of patients received bilateral transverse abdominal plane blocks (TAPB) combined with rectus abdominis sheath blocks (The Anesthesia flow chart is shown in Figure 1).

Postoperative analgesic treatment plan: Sufentanil 5 μg was given as an analgesic bridge 10 min before the end of surgery. When patients were transferred to the PACU, they were given intravenous self-administered analgesia for 48 h. At a rate of 2 mL/h and a PCA volume of 1 mL/time, all three groups of analgesic pumps included hydromorphone 6 mg, ondansetron 32 mg, and flurbiprofen axetil 200 mg.

### 2.2. Observed indicators

#### 2.2.1. General information

Patient’s age, gender, weight, education, time of surgery, preoperative hemoglobin, and any history of hypertension and diabetes mellitus.

#### 2.2.2. Main indicators

Cognitive function indexes: MMSE and MoCA were used to assess the cognitive capabilities of the three groups 1 day before the procedure (T_0_), 3 days after the procedure (T_6_), and 7 days after the procedure (T_7_). All scales were evaluated in a quiet environment by physicians who were unaware of the patient subgroup and were professionally trained. All scales have been authorized by the Department of Psychosomatic Medicine. The examination includes time orientation, place orientation, attention and calculation, immediate memory, delayed memory, visuospatial, and language, and is scored out of 30 (>17 points for those with less than 1 year of education, >20 points for those with 1–5 years, >22 points for those with 6–9 years, and > 23 points for individuals who have completed more than 9 years of schooling). The 1 standard deviation method was used as a diagnostic criterion for early POCD, in which the standard deviation of the patient’s preoperative MMSE and MoCA scores were calculated separately, and the difference value between each patient’s preoperative and postoperative scores was diagnosed as POCD if there was a decrease of ≥1 standard deviation compared with the preoperative standard deviation of that test.

#### 2.2.3. Secondary indicators

Inflammatory response index and S100β protein: 3 mL of peripheral blood specimens were collected at four time points: 1 day before surgery (T_0_), at the conclusion of surgery (T_4_), 24 h after surgery (T_5_) and 72 h after surgery (T_6_). Following a 10-min centrifugation at 3,000 r/min, the serum was separated. The concentrations of serum tumor necrosis factor-α (TNF-α) and central nervous system specific protein S100β were measured by double antibody sandwich enzyme-linked immunosorbent assay (ELISA). Wuhan Huamei Biological Engineering Co. provided the kit.

The patients’ HR and MAP were recorded at each of these four points: before anesthetic induction (T_1_), after induction (T_2_), 30 min into surgery (T_3_), and at the end of surgery (T_4_). Anesthesia recovery indexes [extubation time, Postanesthesia care unit (PACU) residence time, doses of propofol and remifentanil]. The visual analogue scale (VAS) was used to assess postoperative pain status at 24 h (T_5_), 72 h (T_6_), and 7 days (T_7_), with values ranging from 0 to 10, with higher scores indicating more acute pain. Within 48 h following surgery, keep an eye out for and record any instances of adverse reactions in the three patient groups, such as respiratory depression, hypotension, bradycardia, agitation, drowsiness, nausea and vomiting.

### 2.3. Statistical methods

The data were analyzed using SPSS25.0 statistical software and GraphPad Prism 8.0.2 was used for plotting. The pre-test indicated a 12% POCD incidence in the R group. Based on literature searches, expected the incidence of POCD in group D is 11%, group C is 39%. Assuming that Class I error α was assumed to be bilateral 0.05, the test efficacy (1-β) was assumed to be 0.8 and the lost visit ratio was set to 0.1, resulting in 35 cases in each group. Normal or near-normal measures were expressed as mean ± standard deviation ( $\bar{X}$ ± SD), and one-way analysis of variance was used for the comparison of data between groups, and the Bonferroni test was used for two-way comparison between groups. Comparisons of measures at different time points were analyzed using repeated measures ANOVA, and results of within-subjects effect tests were used for compliance with the sphericity test and multivariate tests for non-compliance with the sphericity test. Data that did not conform to normal distribution were described by median and interquartile spacing, and rank sum test was used for comparison of means between groups. Categorical data were reported as *n* (%), and the Chi-squared test was used to compare count data. A statistically significant difference is shown by *p* < 0.05.

## 3. Results

### 3.1. General conditions

The three groups did not differ in terms of age, gender, BMI, years of education, time of surgery, preoperative hemoglobin, or preoperative comorbidities (Table 1).

**Table 1:** Comparison of general data in each group.

Date	Group R (*n* = 34)	Group D (*n* = 35)	Group C (*n* = 35)	*F*/χ^2^	*p*
Age (year)	70.12 ± 3.57	71.26 ± 3.58	69.69 ± 2.52	2.171	0.119
Male sex (%)	21 (61.8%)	20 (57.1%)	21 (60%)	0.156	0.925
BMI (kg·m^−2^)	21.74 ± 1.51	21.46 ± 1.61	20.99 ± 1.52	2.089	0.129
Education	5.62 ± 2.09	6.43 ± 2.66	5.91 ± 2.29	1.044	0.356
Time of operation (min)	223.24 ± 33.95	216.86 ± 42.58	220.14 ± 46.75	0.204	0.816
Preoperative hemoglobin (g/L)	119.79 ± 12.47	117.89 ± 14.89	116.14 ± 11.19	0.686	0.506
Hypertension	9 (26.5%)	11 (31.4%)	8 (22.9%)	0.659	0.719
Diabetes	5 (14.7%)	7 (20%)	6 (17.1%)	0.339	0.844

### 3.2. Hemodynamic indicators

**Figure 2:**
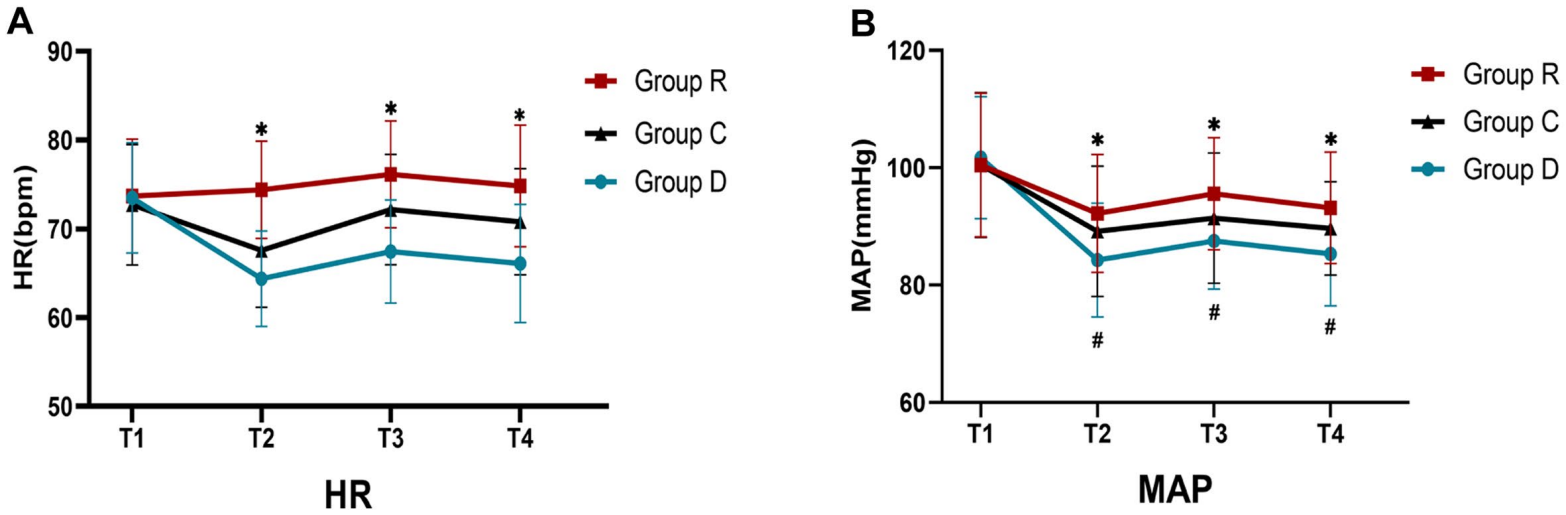
Comparison of HR and MAP in three groups **(A,B)**. ^∗^Comparison with group R, *p* < 0.05; ^#^Comparison with T_1_, *p* < 0.05.

At T_2_, T_3_, and T_4_ time points, group R had greater HR and MAP than group D and C (*p* < 0.05), and from T_1_ to T_2_ time points, HR and MAP dropped considerably in group D and C patients (*p* < 0.05); from T_2_ to T_3_ time points, HR rose in group D and C patients (*p* < 0.05). HR and MAP dropped in all three groups between T_3_ and T_4_ (*p* > 0.05). The incidence of intraoperative hypotension was highest in group D and lowest in group R (*p* < 0.05) (Tables 2–4 and Figure 2).

**Table 2:** Comparison of HR in three groups ( $\bar{X}$ ± SD, bpm).

Indicator	Time	Group R	Group D	Group C	*F*	Effect size	*p*
	T_1_	73.71 ± 6.43	73.49 ± 6.21	72.71 ± 6.82	0.223	0.22 (−3.59 ~ 4.02)	0.80
HR	T_2_	74.41 ± 5.48	64.40 ± 5.38^*,#^	67.57 ± 6.43^*,#^	26.912	10.01 (6.62 ~ 13.40)	<0.001
	T_3_	76.15 ± 6.04^#^	67.46 ± 5.81^a,*,#^	72.17 ± 6.24^a,*^	17.951	8.69 (5.15 ~ 12.22)	<0.001
	T_4_	74.82 ± 6.87	66.11 ± 6.65^*,#^	70.80 ± 5.97^a,*^	15.499	8.71 (4.90 ~ 12.52)	<0.001

^*^Comparison with group R, *p* < 0.05; ^#^Comparison with T_1_, *p* < 0.05; ^a^Comparison with T_2_, *p* < 0.05; effect size presented as difference in means between groups R and D (95% CI).

**Table 3:** Comparison of MAP in three groups ( $\bar{X}$ ± SD, mmHg).

Indicator	Time	Group R	Group D	Group C	*F*	Effect size	*p*
	T_1_	100.47 ± 12.25	101.71 ± 10.43	100.54 ± 12.35	0.124	−1.24 (−8.10 ~ 5.61)	0.884
MAP	T_2_	92.21 ± 10.04^#^	84.29 ± 9.73^*,#^	89.17 ± 11.12^#^	5.187	7.92 (1.87 ~ 13.96)	0.007
	T_3_	95.59 ± 9.59	87.54 ± 8.20^*,#^	91.40 ± 11.13^#^	5.919	8.05 (2.35 ~ 13.73)	0.004
	T_4_	93.15 ± 9.52^#^	85.31 ± 8.85^*,#^	89.69 ± 7.95^#^	6.883	7.83 (2.68 ~ 12.98)	0.002

*Comparison with group R, *p* < 0.05; ^#^Comparison with T_1_, *p* < 0.05; effect size presented as difference in means between groups R and D (95% CI).

**Table 4:** Comparison of the incidence of intraoperative hypotension in three groups [*n* (%)].

Indicator	Group R (*n* = 34)	Group D (*n* = 35)	Group C (*n* = 35)	χ^2^	Effect size	*p*
hypotension	2 (5.9%)	15 (42.9%)^*^	5 (14.3%)	15.628	0.37 (0.19 ~ 0.55)	<0.001

*Comparison with group R, *p* < 0.05; effect size presented as difference in rates between groups R and D (95% CI).

### 3.3. Comparison of anesthesia indicators

**Figure 3:**
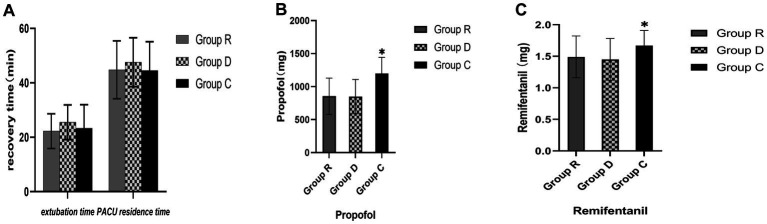
Comparison of anesthesia indicators in three groups **(A-C)**. ^∗^Comparison with group R, *p* < 0.05.

Extubation and PACU residence times were not significantly different between groups R, D, and C (*p* > 0.05), and neither were the propofol and remifentanil dosages between groups R and D (*p* > 0.05). However, the propofol and remifentanil dosages in groups R and D were lower than those in group C, and the difference was statistically significant (*p* < 0.05) (Table 5 and Figure 3).

**Table 5:** Comparison of anesthesia indicators in three groups ( $\bar{X}$ ± SD).

Indicator	Group R	Group D	Group C	*F*	Effect size	*p*
Extubation time (min)	23.34 ± 8.61	25.51 ± 6.37	22.32 ± 6.40	1.769	−3.19 (−7.42 ~ 1.03)	0.176
PACU residence time (min)	44.76 ± 10.65	47.57 ± 8.99	44.54 ± 10.60	0.974	−2.81 (−8.73 ~ 3.11)	0.381
Propofol dosage (mg)	854.41 ± 275.34	847.71 ± 260.33	1200.57 ± 244.13^*^	20.977	6.70 (−145.77 ~ 159.17)	<0.001
Remifentanil dosage (mg)	1.49 ± 0.33	1.45 ± 0.33	1.67 ± 0.24^*^	5.319	0.04 (−0.13 ~ 0.22)	0.006

*Comparison with group R, *p* < 0.05; effect size presented as difference in means between groups R and D (95% CI).

### 3.4. Comparison of TNF-α and S100β

**Figure 4:**
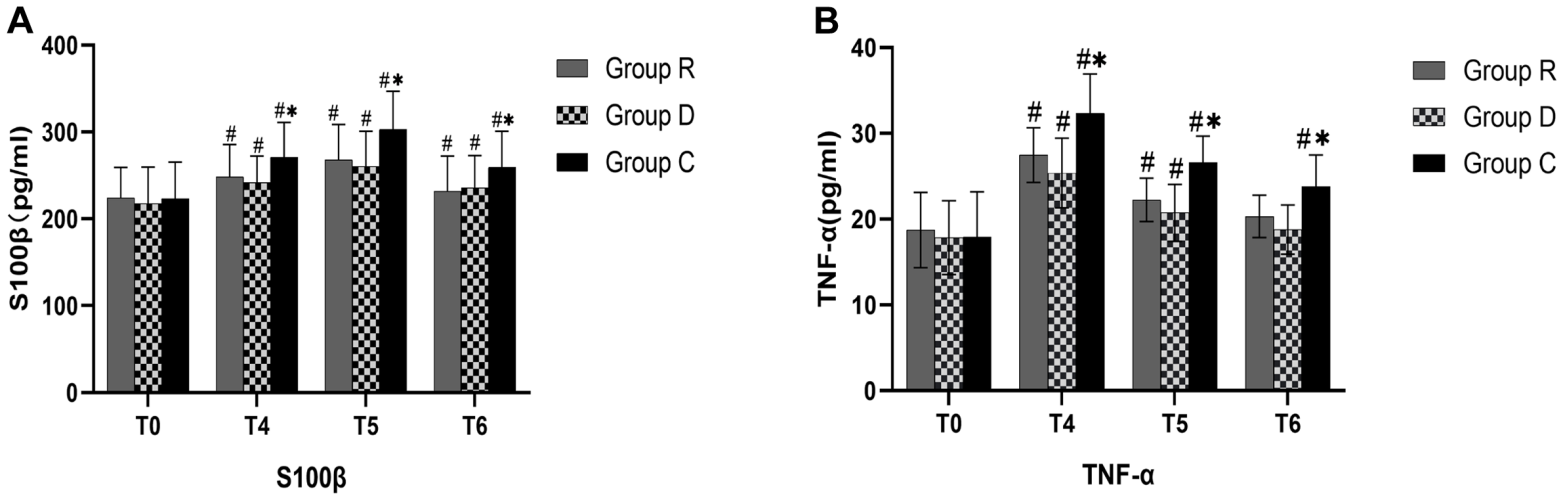
Comparison of S100β and TNF-α in three groups **(A,B)**. ^∗^Comparison with group R, *p* < 0.05; ^#^Comparison with T_0_, *p* < 0.05.

S100β and TNF-α concentration between groups R and D at each time point were not statistically significant (*p* > 0.05). At times T_4_, T_5_, and T_6_, group C had higher S100β and TNF-α concentrations than did groups R and D. These differences were statistically significant (*p* < 0.05) (Tables 6, 7 and Figure 4).

**Table 6:** Comparison of S100β in three groups ( $\bar{X}$ ± SD, pg/mL).

Indicator	Time	Group R	Group D	Group C	*F*	Effect size	*p*
	T_0_	224.03 ± 35.19	217.43 ± 42.06	223.00 ± 42.07	0.274	6.60 (−16.82 ~ 30.02)	0.761
S100β	T_4_	248.24 ± 37.23^#^	241.63 ± 30.51^#^	270.83 ± 40.12^*,#^	6.260	6.61 (−14.59 ~ 27.81)	0.003
	T_5_	267.96 ± 40.58^#^	260.40 ± 40.40^#^	302.83 ± 43.96^*,#^	10.292	7.56 (−16.88 ~ 32.00)	<0.001
	T_6_	231.89 ± 40.37^#^	235.53 ± 37.31^#^	259.26 ± 41.42^*,#^	4.863	−3.63 (−26.92 ~ 19.66)	0.01

*Comparison with group R, *p* < 0.05; ^#^Comparison with T_0_, *p* < 0.05; effect size presented as difference in means between groups R and D (95% CI).

**Table 7:** Comparison of TNF-α in three groups ( $\bar{X}$ ± SD, pg/mL).

Indicator	Time	Group R	Group D	Group C	*F*	Effect size	*p*
	T_0_	18.74 ± 4.39	17.84 ± 4.30	17.90 ± 5.29	0.398	0.90 (−1.84 ~ 3.65)	0.673
TNF-α	T_4_	27.47 ± 3.18^#^	25.37 ± 4.07^#^	32.35 ± 4.56^*,#^	28.162	2.10 (−0.24 ~ 4.43)	<0.001
	T_5_	22.26 ± 2.53^#^	20.74 ± 3.32^#^	26.57 ± 3.10^*,#^	35.445	1.53 (−0.23 ~ 3.29)	<0.001
	T_6_	20.31 ± 2.46	18.78 ± 2.88	23.79 ± 3.70^*,#^	24.659	1.53 (−0.26 ~ 3.33)	<0.001

*Comparison with group R, *p* < 0.05; ^#^Comparison with T_0_, *p* < 0.05; effect size presented as difference in means between groups R and D (95% CI).

### 3.5. Cognitive function

**Figure 5:**
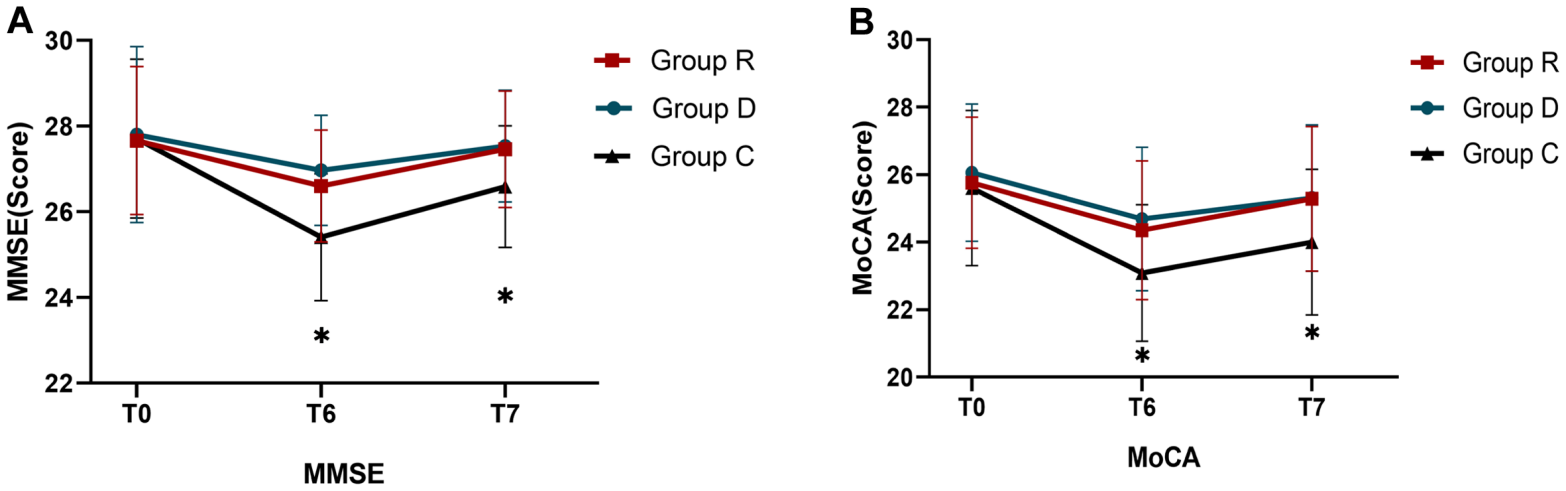
Comparison of MMSE and MoCA scores in three groups **(A,B)**. ^∗^Comparison with group R, *p* < 0.05.

On the first preoperative (T_0_) day for all three groups, there were no statistically significant changes in the MMSE and MoCA test results (*p* > 0.05). The MMSE and MoCA test results for groups R and D at 3 days postoperative (T_6_) and 7 days postoperative (T_7_) were not statistically significant (*p* > 0.05), although both groups scored higher than group C and the difference was statistically significant (*p* < 0.05) (Tables 8, 9 and Figure 5).

**Table 8:** Comparison of MMSE score in three groups ( $\bar{X}$ ± SD).

Indicator	Time	Group R	Group D	Group C	*F*	Effect size	*p*
	T_0_	27.66 ± 1.73	27.80 ± 2.06	27.71 ± 1.85	0.052	0.14 (−0.95 ~ 1.24)	0.949
MMSE	T_6_	26.60 ± 1.31	26.97 ± 1.29	25.41 ± 1.48^*^	12.278	0.37 (−0.42 ~ 1.16)	<0.001
	T_7_	27.46 ± 1.36	27.54 ± 1.31	26.59 ± 1.42^*^	5.156	0.09 (−0.71 ~ 0.88)	0.007

*Comparison with group R, *p* < 0.05; effect size presented as difference in means between groups R and D (95% CI).

**Table 9:** Comparison of MoCA score in three groups ( $\bar{X}$ ± SD).

Indicator	Time	Group R	Group D	Group C	*F*	Effect size	*p*
	T_0_	25.76 ± 1.94	26.06 ± 2.04	25.60 ± 2.30	0.424	0.29 (−0.94 ~ 1.52)	0.655
MoCA	T_6_	24.35 ± 2.06	24.69 ± 2.13	23.09 ± 2.03^*^	5.794	0.33 (−0.88 ~ 1.55)	0.004
	T_7_	25.29 ± 2.14	25.31 ± 2.17	24.00 ± 2.16^*^	4.258	0.02 (−1.24 ~ 1.28)	0.017

*Comparison with group R, *p* < 0.05; effect size presented as difference in means between groups R and D (95% CI).

At 3 days postoperative (T_6_) and 7 days postoperative (T_7_), there was no statistically significant difference (*p* > 0.05) in the incidence of POCD between groups R and D, even though both were lower than the incidence of POCD in group C, which was statistically significant (*p* < 0.05) (Table 10).

**Table 10:** Comparison of the incidence of POCD in each group [*n* (%)].

	*N* (%)	Group R (*n* = 34)	Group D (*n* = 35)	Group C (*n* = 35)	*χ* ^2^	Effect size	*p*
T5	POCD+	6 (17.6%)	6 (17.1%)	14 (40.0%)^*^	6.333	0.005 (−0.173 ~ 0.183)	0.042
T6	POCD+	3 (8.8%)	3 (8.6%)	10 (28.6%)^*^	7.048	0.002 (−0.131 ~ 0.135)	0.029

*Comparison with group R, *p* < 0.05; effect size presented as difference in rates between groups R and D (95% CI).

### 3.6. Comparison of postoperative pain and postoperative adverse effects during the recovery period

**Figure 6:**
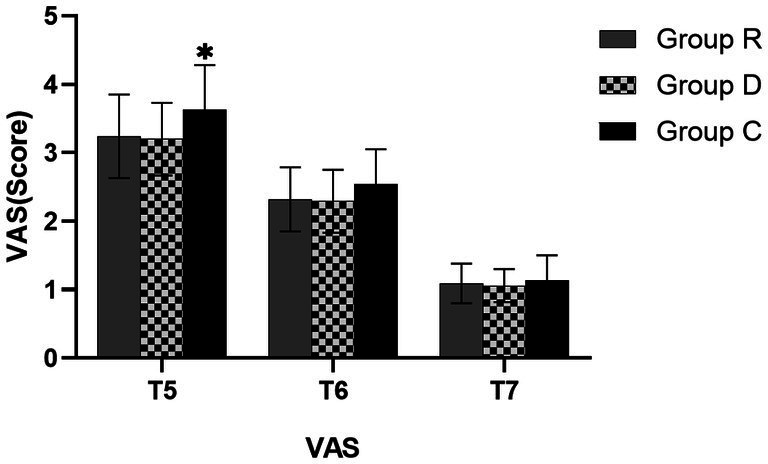
Comparison of VAS scores in three groups. ^∗^Comparison with group R, *p* < 0.05.

There was no significant difference in VAS scores between groups R and D after 24 h postoperatively (*p* > 0.05), although both had lower scores than group C, and the difference was statistically significant (*p* < 0.05). The VAS scores between the three groups at 72 h (T_6_) and 7 days (T_7_) were not statistically significant (*p* > 0.05).In group R, there was no postoperative respiratory depression, hypotension, or bradycardia, and the incidence of postoperative adverse events was considerably lower (*p* < 0.05). The incidence of adverse events was greatest in group C and second highest in group D (Tables 11, 12 and Figure 6).

**Table 11:** Comparison of VAS scores in three groups ( $\bar{X}$ ± SD).

Indicator	Time	Group R	Group D	Group C	*F*	Effect size	*p*
	T_5_	3.24 ± 0.61	3.20 ± 0.53	3.63 ± 0.65^*^	5.555	0.035 (−0.31 ~ 0.38)	0.005
VAS	T_6_	2.32 ± 0.47	2.29 ± 0.46	2.54 ± 0.51	2.920	0.038 (−0.24 ~ 0.32)	0.058
	T_7_	1.09 ± 0.29	1.06 ± 0.24	1.14 ± 0.36	0.747	0.031 (−0.14 ~ 0.20)	0.476

*Comparison with group R, *p* < 0.05; effect size presented as difference in means between groups R and D (95% CI).

**Table 12:** Comparison of the incidence of adverse reactions during the recovery period of each group (%).

Indicator	Group R (*n* = 34)	Group D (*n* = 35)	Group C (*n* = 35)	*χ* ^2^	Effect size	*p*
Respiratory depression	0 (0%)	0 (0%)	3 (8.6%)	4.133	–	0.105
Hypotension	0 (0%)	3 (8.6%)	2 (5.7%)	2.863	–	0.239
Bradycardia	0 (0%)	4 (11.4%)	2 (5.7%)	4.144	–	0.126
Nausea and vomiting	2 (5.9%)	2 (5.7%)	3 (8.6%)	0.285	–	0.867
Agitation	0 (0%)	0 (0%)	3 (8.6%)	4.133	–	0.105
Drowsiness	1 (2.9%)	1 (2.9%)	0 (0%)	1.281	–	0.771
Total incidence	3 (8.8%)	10 (28.6%)^*^	13 (37.1%)^*^	7.736	0.198 (0.02 ~ 0.375)	0.021

*Comparison with group R, *p* < 0.05; effect size presented as difference in rates between groups R and D (95% CI).

## 4. Discussion

Global population aging is accelerating, and an increasing number of senior persons are undergoing surgical therapy. When surgery and anesthesia are performed, the combination of traumatic stress and factors such as CO2 pneumoperitoneum triggers sympathetic excitation in the body and increases the release of catecholamines (Zhang et al., 2019), which can trigger a systemic stress response. Large amounts of inflammatory substances released into the blood enter the brain parenchyma *via* the broken blood–brain barrier, causing POCD. Dexmedetomidine has been demonstrated to block sympathetic nerve activity and lower NF-κB signaling pathway activation, therefore attenuating the inflammatory response and decreasing the incidence of POCD (Zhai et al., 2020). However, when dexmedetomidine is applied, hypotension and bradycardia are more likely to occur, resulting in a hypoperfused brain, and inadequate blood flow to the brain can lead to ischemic damage to brain tissue, which impairs brain function.

### 4.1. Effects of remimazolam on hemodynamic

According to the findings of this investigation, remimazolam induced a slight rise in HR in group R at time T_2_, which is consistent with the findings of Schüttler J in healthy male volunteers with continuous infusion of remimazolam (Schüttler et al., 2020). Among the three groups, group R had the least variation in HR and MAP, the lowest incidence of intraoperative hypotension, and used fewer vasoactive drugs intraoperatively, indicating that remimazolam has the least effect on circulation and can maintain more stable hemodynamics, which is more suitable for elderly patients. In patients undergoing flexible bronchoscopy, remimazolam was shown to have non-inferior efficiency, superior time metrics, and hemodynamic stability when compared to dexmedetomidine (Chen et al., 2022).

### 4.2. Effects of remimazolam on inflammatory response

TNF-α has potent anti-tumor and anti-infection effects at normal concentrations, while excessive TNF-α production can lead to organ dysfunction or death (Boehm et al., 2014). It can cause the body to produce more cytokines (IL-6, IL-8), enhance the production of neurotoxic chemicals, and hasten the destruction of neurological tissue by prompting neutrophils to accumulate (Li et al., 2015). TNF-α was able to reach a peak shortly after the surgery began and subsequently fall, a change that preceded all other inflammatory factors (Bittar et al., 2006). TNF-α concentrations in groups R and C were considerably lower than those in the control group at T_4_, T_5_, and T_6_ time points, owing to remimazolam and dexmedetomidine inhibiting the NF-κB signaling pathway and reducing TNF-α production (Shi et al., 2022), as well as attenuating the associated impaired synaptic plasticity and loss of connectivity in the hippocampus (Alam et al., 2018). The incidence of POCD has decreased.

With the modern notion of speedy recovery, laparoscopy is currently the primary project for treating gastric cancer. The CO2 pneumoperitoneum of laparoscopic surgery can cause a decrease in ventilation as well as a disruption in the internal environment’s acid–base balance, compromising the patient’s lung function and perhaps causing lung damage (Liu et al., 2019). Liu et al. (2021) showed that remimazolam has a role in perioperative lung protection, and it effectively reduced LPS-induced serum pro-inflammatory cytokines production and attenuated multi-organ damage, including lung and liver, which may be related to preventing MAPK and PI3K signaling pathway activation.

### 4.3. Effects of remimazolam on POCD

S100β protein is a calcium-binding protein, mainly produced by astrocytes. When the central nervous system is injured, S100β protein escapes from the damaged cells into the cerebrospinal fluid and then into the blood through the damaged blood–brain barrier (Michetti et al., 2019). Tan et al. (2021) discovered that POCD development was strongly connected with postoperative S100β expression, and hence S100β protein is frequently utilized as a diagnostic for brain damage (Hu et al., 2023). We discovered that the serum concentration of S100β in groups R and D was significantly lower than in group C at time points T_4_, T_5_, and T_6_, because remimazolam and dexmedetomidine attenuated the inflammatory response, inhibited astrocyte and microglia activation, decreased S100β protein release, attenuated the interaction between neurons and glial cells, and protected brain cells and neural tissue from inflammatory factors, maintains intracranial homeostasis, and played a role in brain protection (Wang N. et al., 2022).

Because a single neurological scale test does not adequately indicate changes in the patient’s cognitive function. The MMSE is the most widely used cognitive test, but suffers from ceiling effects and has low discrimination and sensitivity for diagnosing moderate cognitive decline, especially in highly educated individuals (Ciesielska et al., 2016). The MoCA was developed as a rapid screening tool for moderate cognitive decline in order to get over the limitations of the MMSE (Wang et al., 2015). On postoperative days 3 and 7, substantial improvements in cognitive function were seen in groups R and D compared to group C. Among the subtests, there were significant differences in the results for visuospatial and executive functions, memory, and orientation in group C patients. The reason for the lack of differences in the other subtests may be related to the different cognitive aspects and the difficulty level of the test scales.

### 4.4. Effect of remimazolam on the anesthesia indicators

The findings of this experiment revealed no significant differences in the time to extubation and the length of stay in the PACU between groups R, D, and C. Remimazolam has a terminal half-life of 0.75 h, and the metabolite has no pharmacological effect, therefore adding remimazolam to anesthesia maintenance and discontinuing it 40 min before the end of surgery does not prolong anesthesia waking time. Choi et al. compared total intravenous anesthesia with remimazolam and propofol on quality of postoperative recovery (QoR), and discovered that remimazolam was not inferior to propofol as measured by the QoR-15 score, and that pain intensity and analgesic requirements were lower in the remimazolam group (Choi et al., 2022). Multiple randomized clinical trials have demonstrated remimazolam’s safety and efficacy as a sedative and general anesthetic (Chen et al., 2021; Lee et al., 2022).

### 4.5. Effects of remimazolam on postoperative pain and postoperative adverse reactions

Gastric cancer patients are often in a state of immune suppression during the disease, and immune suppression is more pronounced in the advanced stages of the disease (Hurwitz and Watkins, 2012). The stress response induced by surgical trauma and opioid usage further suppresses the immune system, and side effects such as delayed recovery, respiratory depression, nausea and vomiting raise the risk of anesthesia in patients. Opioids have also been associated with increased cognitive dysfunction, and preclinical trials have shown that opioids can trigger a strong neuroinflammatory response through the Toll-like receptor 4 (TLR4) from being activated, leading to POCD (Muscat et al., 2021). TLR4 expression is significantly increased in the hippocampus of aging rodents compared to young rats, so older adults are more likely to develop POCD (Fonken et al., 2016). Groups R and D dramatically lowered the dosage of propofol and remifentanil in this trial, which might alleviate patients’ immune suppression after surgery, and the incidence of postoperative adverse reactions was greatly reduced. After 24 h postoperatively, groups R and D had lower VAS scores than group C. Postoperative healing was aided by the decrease in postoperative pain. Dexmedetomidine produces analgesia by acting on presynaptic and postsynaptic α2 receptors in the posterior horn of the spinal cord and the nucleus of the central solitary tract, cutting off pain signaling pathways, reducing the release of substance P and other injurious sensory neurotransmitters. Remimazolam, on the other hand, can lower the dose of remifentanil and reduce postoperative pain by inhibiting pro-inflammatory factor production and modulating bradykinin B1 receptors and autophagy to relieve neuropathic pain (Xie et al., 2021). Furthermore, there was no postoperative hypotension or respiratory depression in group R. This might be because remimazolam has the ability to regulate hemodynamics while also providing fast anesthesia and arousal with a less inhibitory impact on breathing.

## 5. Conclusion and outlook

### 5.1. Conclusion

Remimazolam is similarly beneficial as dexmedetomidine in lowering the incidence of early POCD in aged patients after radical gastric cancer resection, probably due to reduced inflammatory response.

### 5.2. Shortcomings and prospects

This study did not conduct long-term follow-up of patients, missing statistics on the incidence of POCD after 7 days following the operation, and only two indicators, S100β and TNF-α, were assessed. Also, larger sample sizes allow for the exclusion of more confounding variables, which makes the results appear more precise. Later the group will expand the sample size, extend the study period, evaluate more specific proteins and inflammatory factors associated with POCD, use a variety of combination scales to assess the occurrence of POCD, increase the sensitivity, and conduct in-depth research on the pathogenesis of POCD.

## Data availability statement

The original contributions presented in the study are included in the article/supplementary material, further inquiries can be directed to the corresponding author.

## Ethics statement

The studies involving human participants were reviewed and approved by Ethics Committee of the First Affiliated Hospital of Nanchang University. The patients/participants provided their written informed consent to participate in this study.

## Author contributions

YL and JM devised the experiments, examined the data, and wrote the paper. The experiments were carried out by ZH and ZW, who also revised the text. All authors contributed to the article and approved the submitted version.

## Funding

This study was supported by a grant (No. 82060217 to JM) from the National Natural Science Foundation of China, Beijing, China.

## Conflict of interest

The authors declare that the research was conducted in the absence of any commercial or financial relationships that could be construed as a potential conflict of interest.

## Publisher’s note

All claims expressed in this article are solely those of the authors and do not necessarily represent those of their affiliated organizations, or those of the publisher, the editors and the reviewers. Any product that may be evaluated in this article, or claim that may be made by its manufacturer, is not guaranteed or endorsed by the publisher.
